# Potentially Inappropriate Medication: A Pilot Study in Institutionalized Older Adults

**DOI:** 10.3390/healthcare12131275

**Published:** 2024-06-26

**Authors:** Amanda Andrade, Tânia Nascimento, Catarina Cabrita, Helena Leitão, Ezequiel Pinto

**Affiliations:** 1 Escola Superior de Saúde, Universidade do Algarve (ESSUAlg), Campus de Gambelas, Edifício 1, 8005-139 Faro, Portugal; aoandrade@ualg.pt (A.A.); tinascimento@ualg.pt (T.N.); cicabrita@ualg.pt (C.C.); 2 Algarve Biomedical Center Research Institute (ABC-RI), Universidade do Algarve, Campus de Gambelas, Edifício 2, 8005-139 Faro, Portugal; hcleitao@ualg.pt; 3 Faculdade de Medicina e Ciências Biomédicas, Universidade do Algarve, Campus de Gambelas, Edifício 2, 8005-139 Faro, Portugal

**Keywords:** drug interactions, institutionalized elderly, medication review, polypharmacy, potentially inappropriate medications

## Abstract

Institutionalized older adults often face complex medication regimens, increasing their risk of adverse drug events due to polypharmacy, overprescribing, medication interactions, or the use of Potentially Inappropriate Medications (PIM). However, data on medication use and associated risks in this population remain scarce. This pilot study aimed to characterize the sociodemographic, clinical and pharmacotherapeutic profiles, and the use of PIM among institutionalized elders residing in Residential Structures for Elderly People (ERPI) in the Faro municipality, located in the Portuguese region of the Algarve. We conducted a cross-sectional study in a non-randomized sample of 96 participants (mean age: 86.6 ± 7.86 years) where trained researchers reviewed medication profiles and identified potentially inappropriate medications using the EU(7)-PIM list. Over 90% of participants exhibited polypharmacy (≥5 medications), with an average of 9.1 ± 4.15 medications per person. About 92% had potential drug interactions, including major and moderate interactions. More than 86% used at least one potentially inappropriate medication, most commonly central nervous system drugs. This pilot study demonstrates that institutionalized older adults may be at high risk of potential medication-related problems. Implementing comprehensive medication review programs and promoting adapted prescribing practices are crucial to optimize medication use and improve the well-being of this vulnerable population.

## 1. Introduction

Advancements in healthcare and medicine have significantly improved quality of life and life expectancy, contributing to an increase in global population ageing. Portugal had a rate of ageing of 183.5% in 2022 [1], with 29.7% of the population aged 60 or over and projected to rise to around 40.3% by 2050 [2]. Globally, according to the United Nations, the number of elderly people aged 60 or over could double by 2050, from 962 million in 2017 to 2.1 billion in 2050 [3].

As physical and cognitive capacities decline with age, the elderly face diminished self-care abilities [4,5]. In Portugal, Residential Structures for the Elderly (ERPI) play a crucial role by providing health care, psychosocial support, and promoting autonomy and protection for the elderly [6]. According to Portuguese legislation, ‘a residential structure for the elderly is an establishment for collective accommodation, for temporary or permanent use, in which social support activities are carried out and adequate care is provided, adjusted to the needs of the elderly and their families’. This care includes, for example, food appropriate to needs, personal hygiene care, clothing care, nursing care and access to health care, or the administration of medication when prescribed [6]. ERPI are state- or privately funded institutions, and are widely known as nursing homes.

Most elders have chronic illnesses that contribute to a decrease in autonomy and self-care ability, which results in the prescription of various pharmacological therapies and, consequently, polymedication [7].

There is no universal definition of polymedication, but it is commonly understood to involve the concurrent use of five or more medications [8], as this increases the risk of adverse reactions and drug interactions and can lead to negative and unexpected clinical results [9]. In elderly patients, polymedication is often categorized into two large groups: (i) polymedication, when five to nine drugs are taken simultaneously; and (ii) excessive polymedication, when 10 or more drugs are taken [9,10,11,12,13,14]. In the elderly population, the number of polymedicated and excessively polymedicated patients is 31.5% and 20.1%, respectively [11]. In addition to drug interactions and the consequences they entail, polymedication leads to significant economic and clinical problems, including increased risk of falls, hospitalizations, and healthcare costs [13,14].

Promoting the appropriate use of medications through multidisciplinary teams, reviewing pharmacotherapy, and tailoring medication to individual needs could help decrease mortality and the risks associated with polypharmacy [8,15]. In environments where elderly individuals receive care from specialized institutions or qualified health professionals, the risks of medication-related problems are notably decreased [16,17]. The elderly population is diverse, with varying medical conditions and degrees of physical, cognitive, and metabolic decline, making it challenging to identify and prevent adverse drug reactions [18]. Thus, individual needs, pharmacokinetic and pharmacodynamic changes, polymedication, and multimorbidity are risk factors that should be considered in pharmacotherapeutic analyses in this population [19].

The use of Potentially Inappropriate Medications (PIM) represents an increase in the complexity of pharmacotherapy in the elderly. In addition to anatomophysiological and metabolic changes, interactions, and risk for adverse reactions associated with any medication, research shows that there are medications that are considered inappropriate for elders [20]. PIM are drugs where the associated risks outweigh the clinical benefits, putting the patient at risk and prone to serious health risks [21,22]. Analyses of routine prescriptions and medication use in hospitals and nursing homes have shown that the prevalence of PIM use by elderly patients is high [23,24].

The use of PIM is associated with polymedication and with the incidence of adverse effects [23,25,26,27]. Although PIM should be avoided in the elderly, sometimes their use is necessary, due to the lack of effective alternatives or when their discontinuation could cause more harm. When used inappropriately, without following medical recommendations and without proper monitoring, PIM can lead to increased hospitalizations, falls, adverse reactions, and mortality among the elderly [28,29].

The pharmacotherapeutic regime for the elderly is complex, influenced by multimorbidity and age-related factors that contribute to polymedication and associated medication problems [17]. In addition to the extensive medical care that is already necessary at this stage of life, pharmaceutical care and medication review can favor quality of life and patient safety [30]. According to the Pharmaceutical Care Network Europe (PCNE), medication review “…is a structured assessment of a patient’s medicines with the aim of optimizing the use of medicines and improving health outcomes. This involves detecting medication-related problems and recommending interventions” [31]. Medication review leads to better clinical results from therapeutic practice and is therefore an extremely important procedure, especially for elderly polymedicated patients with multiple health issues [32]. In Portugal, there are no legal obligations to conduct mandatory medication reviews in ERPI. This type of service can be provided by the pharmacy that dispenses the medicines for the institution or by a nurse working for the ERPI, but it is not mandatory.

This pilot study aimed to characterize the sociodemographic, clinical and pharmacotherapeutic profile, and the use of PIM of institutionalized elders residing in two ERPI in the Faro municipality in the Portuguese region of the Algarve.

## 2. Materials and Methods

We conducted a cross-sectional descriptive study in two ERPI located in the municipality of Faro, Portugal. ERPI selection and invitation were decided by convenience, without additional criteria apart from distance. The study included a total of 96 elderly people aged 65 or over, taking at least two medications and who consented the collection of their clinical and medication data, and summary clinical and demographic characteristics.

Analysis of the existing pharmacotherapeutic profile for each participant allowed us to retrieve the names of the medicines (prescription and nonprescription) and food supplements used, pharmaceutical form, dosage, and dosage regimen. A Type 2B medication review was carried out according to the Pharmaceutical Care Network Europe criteria [31]. This type of review is an intermediate level of medication review performed when both the patient’s medication history and relevant medical information are available, allowing for a more in-depth analysis than a simple review of the patient’s medication history alone (Type 1). It allows us to identify potential drug interactions, side effects, dosage issues, and adherence problems, as well as drug-food interactions and issues with the effectiveness of the medication [31].

All medications were organized according to the Anatomical Therapeutic Chemical Code (ATC), level 3 [33]. The use of ≥5 medications was considered “polymedication” and the use of ≥10 medications was considered “excessive polymedication”. Individuals with less than five medications were considered “not polymedicated” [11].

Potential medication interactions were classified using the Lexidrug^TM^ Drug Interaction Module tool from the UpToDate platform. This tool classifies interactions into five different categories according to the associated risk: type A (no potential pharmacokinetic or pharmacodynamic interactions; no known interaction), type B (little to no evidence of clinical concern; no action needed), type C (clinically significant interactions; monitor therapy), type D (clinically significant interactions and risk-benefit must be assessed; consider therapy modification), and type X (clinically significant interactions where the risks outweigh the benefits; avoid combination) [34].

Potentially inappropriate medicines for the elderly were considered according to the Portuguese operationalization [35] of the European EU(7)-PIM List [22], and the complexity of the pharmacotherapeutic regime was assessed using the criteria established by the Medication Regimen Complexity Index tool (MRCI) [36]. The MRCI score is calculated from three main subtotals, each contributing to the overall complexity. Subtotal A refers to the pharmaceutical form of the medication (e.g., tablet, injection, inhaler), as different forms may have different complexities. Subtotal B refers to the dosage frequency; more frequent dosing schedules increase the complexity. Subtotal C refers to the instructions and additional precautions for each medication, such as dietary restrictions or to avoid certain activities. Points are added with increased complexity added by the form, with increased frequency of dosing, and with additional specific instructions. The MRCI score is the sum of all subgroup scores and a total score >16.5 can be interpreted as high pharmacotherapeutic complexity [37].

Statistical analysis was carried out using IBM SPSS Statistics software for Macintosh, version 29.0. Armonk, NY, USA: IBM Corp.

Data were described using absolute and relative frequencies, mean (M), median (Mdn), standard deviation (SD), and interquartile range (IQR). We used the Kolmogorov–Smirnov test to assess adherence to the Normal distribution. All variables in our study were either categorical or with a non-Normal distribution. Thus, group comparisons were made using Kruskal–Wallis, chi-square, and Mann–Whitney’s tests. Correlations were computed using Spearman’s correlation coefficient. Statistical significance for all procedures was set at 0.05.

## 3. Results

### 3.1. Sociodemographic and Pharmacotherapeutic Characterization

There were 96 patients included in this study, all aged 65 or over and residing in care institutions for the elderly. Mean age was 86.6 ± 7.86 years, ranging from 68–109 years, with only 9.4% aged between 65–74 and 66.7% aged 85 or above. Most participants were women (68.8%). We found significant gender differences in age (*p* = 0.044), with women (87.9 ± 6.9) being older than men (83.7 ± 9.04).

Mean number of medications used was 9.1 ± 4.15, ranging from 2 to 24, and 90.6% of participants (*n* = 87) were considered polymedicated (≥5 medications). Of the polymedicated, 47.1% (*n* = 41) were excessively polymedicated (≥10 drugs). Only 9.4% of patients (*n* = 9) had a pharmacotherapeutic profile with fewer than five drugs. Of the patients identified as polymedicated, 60.4% were aged 85 or over and 62.5% were female. The mean number of medications in the participants without polypharmacy was 3 ± 0.87, while those with polypharmacy showed a significantly higher (*p* < 0.001) mean of 9.8 ± 3.83 medications.

According to the MRCI score, pharmacotherapeutic complexity showed a mean score of 19.2 ± 9.90, ranging between 5–55 points. Subtotals A, B, and C had means of 3.2 ± 2.39, 10.3 ± 5.26, and 5.7 ± 3.95 points, respectively. Table 1 presents age and selected data on medication use. No statistically significant differences in medication use were found between male and female participants (*p* > 0.05).

MRCI score showed a strong positive correlation with the total number of medications used (R = 0.873; *p* < 0.001) and a negative correlation with age (R = −0.219, *p* = 0.032), suggesting that younger patients and those that use more medications have a higher pharmacotherapeutic complexity. The statistically significant correlation of MRCI with age does not hold up when considering patients aged 65–74 years (R = 0.156, *p* = 0.688; *n* = 9) or ≥85 years (R = −0.005, *p* = 0.967; *n* = 64). This correlation is still significant in patients aged 75–84 years (*n* = 23), but suggests a positive association, i.e., higher complexity in older patients (R = 0.550, *p* = 0.007). After adjusting for the number of medications, the correlation between MRCI and age was not significant (R = 0.038, *p* = 0.867) in this demographic. This suggests that the number of medications may have confounded the initial association, and that factors other than age, such as clinical condition, could be contributing to pharmacological complexity. Table 2 presents the correlation matrix between MRCI and other variables.

Of the participants, 91.7% (*n* = 88) had at least one potential drug interaction, with a mean of 10.7 ± 10.55 interactions per participant, ranging between 0–57. At least one potential interaction of each of the five types considered by the Lexidrug^TM^ tool was detected. Type C interactions were the most prevalent (86.5%; *n* = 83) and had the highest mean occurrence per patient (7.93 ± 7.68). Type A interactions, those with no potential pharmacokinetic or pharmacodynamic interactions, were seen in only two patients (2.0%), with a mean of 0.1 ± 0.92 interactions. The most serious and clinically significant interactions (type X), where the risks outweigh the benefits and that should be avoided, were identified in 13 patients (13.5%), with a mean of 0.19 ± 0.53. One of the patients presented those three interactions of this type simultaneously. Our data also show that 59.4% (*n* = 57) of the sample had at least one type B interaction, with an average of 1.34 ± 1.89 of these type of interactions per participant. Type D interactions were identified in 40.6% (*n* = 39) of patients, with an average of 1.10 ± 2.02 interactions. The number of interactions is positively correlated with pharmacologic complexity (R = 0.692, *p* < 0.001). All interaction types apart from type A were also correlated with pharmacologic complexity (*p* < 0.001).

### 3.2. Potentially Inappropriate Medication Characterization

Our data show that 86.5% (*n* = 83) of the patients used at least one PIM (with a mean of 2.2 ± 1.56 PIM per participant, ranging between 0–6), 67.5% (*n* = 56) were women, 63.9% (*n* = 53) were aged 85 or over, and 92.8% (*n* = 77) had some kind of potential drug interaction and were polymedicated (≥5 medications). More than half of the patients who used any PIM (54.2%, *n* = 45) had high pharmacotherapeutic complexity scores (MRCI > 16.5 points). PIM was positively correlated with MRCI score (R = 0.412, *p* < 0.001), but no statistically significant difference in total MRCI score (*p* = 0.368) was found when comparing patients who used PIM and those who do not. Nevertheless, subtotal B from the MCRI score was different in these patients (*p* = 0.047).

No statistically significant differences in age, gender, polymedication, and interactions were observed when comparing patients who used PIM and those who do not (*p* > 0.05). Table 3 presents the comparisons between patients using PIM and those who do not use PIM.

PIM from the EU(7)-PIM list were classified according to their ATC. In total, 209 PIM were identified and distributed among 7 ATC groups (A, B, C, G, M, N, and R). Of the total number of PIM, 49.8% (*n* = 104) belong to central nervous system medications (ATC N) and 29.2% (*n* = 61) to alimentary tract and metabolism medications (ATC A). Within these two groups, the use of anxiolytics (ATC N05B) and drugs to treat peptic ulcers and gastroesophageal reflux (ATC A02B) stood out, accounting for 23.4% (*n* = 49) and 23.9% (*n* = 50), respectively, of the total number of PIM.

Mean PIM in ATC G is significantly lower (*p* = 0.024) in patients ≥85 years old (M = 0.19 ± 0.5) than in younger patients (M = 0.44 ± 0.67). Similarly, patients ≥85 years old also have lower (*p* < 0.001) PIM in ATC N (M = 3 ± 1.79) than younger patients (M = 4.8 ± 2.55). Polymedicated patients have a higher (*p* = 0.009) mean number of PIM in ATC A (M = 1.6 ± 1.25) than non-polymedicated patients (M = 0.6 ± 0.53), and patients with a high pharmacotherapeutic complexity have a higher (*p* < 0.001) PIM in ATC C (M = 2.5 ± 1.7) than all other patients (M = 1.3 ± 1.1).

Of the patients who use PIM, 53.1% (*n* = 51) use at least one from ATC A and 62.5% (*n* = 60) use at least one from ATC N.

## 4. Discussion

In this cross-sectional study, we reviewed the medication of elders institutionalized in two ERPI. Our sample primarily consisted of individuals over 80, predominantly women, aligning with demographics reported in similar studies from the United States, Spain, Portugal, France, and Colombia [38,39,40,41]. This demographic trend underscores the critical need for targeted pharmacotherapy management, particularly given the elderly’s propensity for chronic diseases and the consequent polymedication [19,42].

Our data show the use of 9.13 ± 4.15 medicines, ranging from 2 to 24, in accordance with previous research in Portugal and France suggesting high medication usage among the institutionalized elderly. The Portuguese study reports a mean use of 10.1 ± 3.9 medications, ranging from 5 to 28 [43], and the French study reports a mean of 8.1 ± 4.0, ranging from 1 to 20 medications [44]. However, the tools used to analyze the PIM were different, which may reflect differences in the results. Other studies reflect slight variances in medication usage, emphasizing the complexity of pharmacotherapy in this demographic [23,43].

Pharmacotherapeutic complexity can be assessed considering numerous factors, but the complexity index is one of the few methodologies capable of numerically quantifying this criterion [36,45,46] and is validated for Portuguese [36]. Studies from Brazil and Portugal reported similar MRCI means to the one in our data, while Australian studies found higher complexity [47,48,49,50]. The MRCI is instrumental in gauging the challenge each pharmacotherapeutic profile presents, and our findings corroborate a direct correlation between MRCI values, the number of medications, and patient age [49,51].

One of the decisive points for a good therapeutic review is the identification of potential drug interactions that could jeopardize clinical conditions and the treatment of pathological conditions [30,52]. Studies carried out in this area show different results in terms of the percentage of institutionalized elderly people with potential drug interactions. Research carried out in Brazil, Greenland, and Spain revealed 25.4%, 61.0%, and 81.1% of patients, respectively, with potential interactions [53,54,55], although our data showed more than 90% of the sample with potential interactions, underscoring the critical need for vigilant drug management in this population. The differences found in potential drug interactions may be due to the assessment tool used. The literature shows that, depending on the tool used to assess potential drug interactions, the results can be different [56,57]. However, Lexidrug^TM^ is considered a tool with excellent performance and comprehensiveness in identifying potential drug interactions [58], as evidenced by original research comparing several tools. Kheshti et al. compared the accuracy and comprehensiveness of five drug interaction software programs (Lexi-Interact, now Lexidrug^TM^, Micromedex Drug Interactions, iFacts, Medscape, and Epocrates) using Stockley’s Drug Interaction and databases, including PubMed, Scopus, and Google Scholar, as a reference. A total of 360 prescriptions, randomly selected from 2 pharmacies, were analyzed by an experienced clinical pharmacist. Lexidrug^TM^ proved to be the most accurate software and the second most comprehensive [59]. Hecker et al. compared three drug interaction databases for screening severe interactions in patients with multiple sclerosis. The Stockley’s, Drugs.com, and MediQ databases were compared using the medication of 627 patients followed in the neurology department of hospitals in Germany. Although Stockley’s was the database that identified the most potential severe interactions (37.4%), the information obtained between the databases was considered heterogeneous [60]. The differences found in the various databases for analyzing potential drug interactions may be due to the sources of information and levels of evidence considered in each one. Given the diversity of results obtained, it is recommended in clinical practice to use more than one source to assess potential drug interactions [57,59,60].

The various existing tools for assessing PIM make it difficult to compare our data with results from the literature. For instance, a study comparing three PIM identification tools in the same sample found variability between 35.3% and 96.3% in the detection rates of participants with at least one inappropriate medication. The PRISCUS list had the lowest identification rate (35.5%), followed by the Beers criteria (90.8%); the tool with the highest identification rate (96.3%) was the STOPP Criteria [61]. This variability points to a generally high tendency toward prescribing PIM in institutionalized settings, confirmed by our findings (86.5%) and literature suggesting prevalence rates from 79.1% to 96.8% [23,62,63].

Population-based studies involving both institutionalized and non-institutionalized elders reported lower PIM usage (24.1% and 40%) likely due to reduced PIM consumption among the non-institutionalized [64,65,66,67]. The organization and care provided in institutions, the pathologies diagnosed, the greater number of drugs, and the classes of drugs prescribed in institutions are pointed to as possible reasons for the differences found in the number of potentially inappropriate drugs between institutionalized and non-institutionalized elderly people [66,67]. A study comparing institutionalized elders and elderly outpatients showed that 82.4% of institutionalized participants were prescribed PIM, while this prevalence was 34.4% in outpatients [68]. This trend was also reported by a Swedish study, which suggested institutionalization as a potential risk factor for the use of PIM and proposing greater attention and care when prescribing drugs for institutionalized elderly people [69]. Contrary results were described in a study carried out in Ontario, Canada, where the number of potentially inappropriate medications in institutionalized elderly people was lower than that found in elderly people in the community. However, clinical pharmaceutical services, such as medication review, are mandatory in institutions for the elderly, which may justify the results found [70].

Another study, carried out in Portugal, also compared three lists for identifying potentially inappropriate medicines (Beers 2019, Screening Tool of Older People’s Potentially Inappropriate Prescriptions (STOPP) version 2, and EU(7)-PIM list) in elderly patients followed in primary healthcare. Their results showed little agreement between the lists used, with the greatest agreement found between the EU(7)-PIM list and the Beers 2019 criteria. The Beers 2019 criteria were the most sensitive in detecting PIM in these patients [71]. The same low level of agreement was found between the three lists applied to 616 elderly patients admitted to the internal medicine department of a Portuguese hospital. In this study, the highest agreement was found between the Beers 2019 criteria and the STOPP criteria [72]. A study carried out in Brazil showed moderate to high agreement between four different tools (Beers-2015, STOPP-2015, EU(7)-PIM list, and Taiwan criteria). The EU(7)-PIM criteria showed a high level of sensitivity but a low level of specificity, while the Beers criteria showed a more balanced sensitivity/specificity ratio [73].

The discrepancy in results may be due to the data required to identify PIM in the various tools. While identification with EU(7)-PIM requires information on dosage or duration of treatment [35], application of the Beers criteria requires additional information such as diagnosis of pathologies or information on renal function [74,75]. For the STOPP criteria, clinical and laboratory information is also required, in addition to the pharmacotherapeutic profile of the elderly [76]. These are factors that can not only influence the results obtained but also make it difficult to evaluate PIM in different contexts, as the data available for collection may have gaps that prevent the tools from being applied.

Moreover, our study aligns with other research indicating that female and polymedicated elderly patients are more likely to receive at least one PIM [62,65,67].

In Portugal, a national study suggested a significant consumption of PIM, with 1232 billion defined daily doses prescribed, resulting in an overall prevalence of 9.20%. This study showed a high consumption of PIMs, particularly anxiolytics and antidepressants, despite the implementation of national measures to prescribe these medicines. In the Algarve region, there seems to be an upward trend in the number of potentially inappropriate medicines [77]. This reality shows that it will be important to implement different measures to improve the safe use of medicines in institutionalized elderly people. Medication reviews could be an important service to implement [66].

Regarding ATC classification of PIM, our data are in accordance with the literature. A study on institutionalized elderly with dementia across eight countries identified major PIM within psycholeptics and drugs treating acid-related disorders (ATC N05 and ATC A02, respectively) [78]. A Portuguese study with institutionalized patients, which also used the EU(7)-PIM List, revealed a similar trend: proton pump inhibitors (A02BC) were the most prescribed PIM (25.8%), followed by anxiolytics (N05BA, 20.5%) [23]. Another study carried out using three different tools to identify PIM reported significant prescription of proton pump inhibitors and drugs associated with the nervous system among the PIM detected [61]. As mentioned above, it is to be expected that drugs from the ATC A and N groups are the most prescribed PIM, since they also correspond to the therapeutic groups more often used by institutionalized elderly people.

Regarding the number of medications used and the prescription of PIM, our results are also in accordance with the literature, such as a population-based study suggesting a significant association between these two variables (*p* < 0.001) and higher mean medications in use by patients with PIM (7.7 vs. 5.6) [63]. Furthermore, regarding pharmacotherapeutic complexity, the literature suggests that the presence of PIM is also associated with a likelihood of higher MCI in institutionalized elderly (*p* < 0.001) [48].

Our findings suggest that elderly patients below 85 years old use more PIM, as a study reporting a similar trend for patients under 70 [79]. This may reflect a tendency to prescribe fewer medications to older individuals [80,81].

It may also reveal a trend towards deprescribing in elderly individuals, particularly those who are polymedicated. Although slowly, the process of deprescribing in the elderly is beginning to emerge in clinical practice, and it has many advantages, especially in polymedicated elderly people [82]. This practice can bring benefits to institutionalized elderly people, although it requires a multidisciplinary approach that can be difficult to implement [83,84,85]. A longitudinal multicenter cohort study, which included 1843 elderly people from 7 European countries and Israel, showed that after 1 year of follow-up, 35.7% of the participants had been subjected to deprescribing. Although it was not statistically significant, the presence of a pharmacist on staff was associated with a higher rate of deprescribing [86]. In institutionalized elderly people, pharmacist-led deprescribing recommendations can have a significant impact on reducing the number of inappropriate medicines consumed by the elderly and are accepted by the doctor in a multidisciplinary work [87]. Furthermore, there are still several barriers identified by those involved that can hinder the implementation of this practice [88]. The patient’s individual characteristics also play an important role in the deprescribing process [89]. The patient’s level of dependence significantly influences doctors’ attitudes to deprescribing, as does life expectancy or quality of life. The benefits and risks of medication are also considered very important criteria in the practice of deprescribing, as are the existence of guidelines and tools to support deprescribing [90].

Despite the barriers, medication review oriented towards deprescribing is important in institutionalized elderly people because, according to a systematic review and meta-analysis, it significantly reduces the number of potentially inappropriate medications, as well as the number of falls and even the risk of death [91].

We identify some limitations in our study. Even if this was not an objective in our study, our data do not allow us to conduct an in-depth analysis of the clinical variables and of the assumptions and reasons for the prescription of some medications. The non-random nature of the sample implies limited external validity for our results and our findings should be considered according to context. We cannot extrapolate for the institutionalized elderly population in the Algarve region, but this pilot study allowed us to assess the applicability of the data collection tools on this population and to carry out a type 2B medication review.

## 5. Conclusions

This pilot study shows show that institutionalized older adults may be at high risk of potential medication-related problems, and highlighted several key challenges associated with pharmacotherapeutics in the elderly, such as the excessive number of medicines prescribed, high therapeutic complexity, the frequent use of PIM, and a high prevalence of potential drug interactions.

While scientific research demonstrates that medication review is a crucial intervention for identifying and addressing medication-related issues, especially in the elderly who often take multiple medications, our results suggest that medication review may remain underutilized within care institutions. This underscores a critical gap in practice that needs addressing. Enhancing awareness and understanding of medication review’s benefits among healthcare providers and institutional managers is essential. A broader dissemination of this practice should be considered in order to integrate it as a standard component of elderly care.

## Figures and Tables

**Table 1:** Age and medication use for all participants and by gender.

Characteristics (M ± SD)		Total Sample	Females	Males	*p*-Value
		(*n* = 96)	(*n* = 66)	(*n* = 30)	
Age (years)		86.6 ± 7.86	87.9 ± 6.94	83.7 ± 9.04	0.044
No. of medications (total)		9.1 ± 4.15	9 ± 4.25	9.3 ± 4	0.565
5–9 medications; *n* (%)		46 (47.9)	33 (50)	13 (43.3)	0.831 ^c^
≥10 medications; *n* (%)		41 (42.7)	27 (40.9)	14 (46.7)	
Interactions (total)		10.7 ± 10.54	10.7 ± 10.92	10.5 ± 9.85	0.997
	Type A	0.1 ± 0.92	0.1 ± 1.11	0 ± 0.18	0.576
	Type B	1.3 ± 1.89	1.6 ± 2.1	0.9 ± 1.22	0.077
	Type C	7.9 ± 7.68	7.8 ± 7.75	8.2 ± 7.64	0.809
	Type D	1.1 ± 2.02	1.1 ± 2.05	1.1 ± 1.97	0.775
	Type X	0.2 ± 0.53	0.1 ± 0.45	0.3 ± 0.66	0.054
MRCI score (total)		19.2 ± 9.9	18.6 ± 10.02	20.4 ± 9.68	0.213
	Subtotal A	3.2 ± 2.39	2.9 ± 2.14	3.9 ± 2.76	0.084
	Subtotal B	10.3 ± 5.26	10.2 ± 5.53	10.7 ± 4.68	0.342
	Subtotal C	5.7 ± 3.95	5.6 ± 4	5.9 ± 3.91	0.625
PIM-EU List		2.2 ± 1.56	2.1 ± 1.6	2.3 ± 1.47	0.395

Characteristics are presented as mean (M) and standard deviation (SD), with noted exceptions; MRCI—Medication Regimen Complexity Index; PIM—Potentially inappropriate medications; Interactions classified according to Lexidrug^TM^ Drug Interaction Module; Gender differences computed with Mann-Whitney’s test except in: ^c^—chi-square test.

**Table 2:** Correlations with Medication Regimen Complexity Index (MRCI).

Variable	Correlation with Total MRCI Score			
	All (*n* = 96)	65–74 (*n* = 9)	75–84 (*n* = 23)	85+ (*n* = 64)
Age	−0.219 *	0.156	0.550 **	−0.005
MRCI A	0.598 **	0.322	0.526 **	0.592 **
MRCI B	0.910 **	0.987 **	0.924 **	0.898 **
MRCI C	0.859 **	0.657	0.896 **	0.835 **
No. of medications	0.873 **	0.928 **	0.887 **	0.866
Interactions (total)	0.692 **	0.450	0.854 **	0.608 **
Type A	0.079	0.138		0.031
Type B	0.506 **	0.479	0.480 *	0.579 **
Type C	0.639 **	0.392	0.885 **	0.542 **
Type D	0.482 **	0.538	0.591 **	0.386 **
Type X	0.387 **	0.143	0.396	0.361 **
PIM	0.412 **	0.658	0.707 **	0.115

PIM—Potentially inappropriate medications; Interactions classified according to Lexidrug^TM^ Drug Interaction Module; *—significant correlation at *p* < 0.05; **—significant correlation at *p* < 0.001.

**Table 3:** Comparisons between patients with and without potentially inappropriate medications (PIM).

Characteristics (M ± SD)		Without PIM	Using PIM	*p*-Value
		(*n* = 13)	(*n* = 83)	
Age (years)		88.8 ± 7.8	86.2 ± 7.85	0.245
Gender	Female; *n* (%)	10 (76.9)	56 (67.5)	0.494 ^c^
	Male; *n* (%)	3 (23.1)	27 (32.5)	
No. of medications		6.8 ± 3.26	9.5 ± 4.18	0.063
5–9 medications; *n* (%)		6 (46.2)	40 (48.2)	0.172 ^c^
≥10 medications; *n* (%)		4 (30.8)	37 (44.6)	
Interactions (total)		6.6 ± 5.33	11.3 ± 11.03	0.187
	Type A		0.1 ± 0.99	0.574
	Type B	0.6 ± 96	1.5 ± 1.98	0.085
	Type C	5.6 ± 4.37	8.3 ± 8.03	0.462
	Type D	0.4 ± 0.51	1.2 ± 2.14	0.374
	Type X		0.2 ± 0.56	-
MRCI score (total)		16.2 ± 7.47	19.6 ± 10.19	0.368
	Subtotal A; M ± SD	3.8 ± 2.71	3.1 ± 2.34	0.266
	Subtotal B; M ± SD	7.4 ± 3.49	10.8 ± 5.36	0.047
	Subtotal C; M ± SD	5.1 ± 3.38	5.8 ± 4.04	0.867

Characteristics are presented as mean (M) and standard deviation (SD), with noted exceptions; MRCI—Medication Regimen Complexity Index; Interactions classified according to Lexidrug^TM^ Drug Interaction Module; Group differences computed with Mann-Whitney’s test except in: ^c^—chi-square test.

## Data Availability

Due to privacy restrictions, the data presented in this study are only available on request from the corresponding author.
